# proNGF Involvement in the Adult Neurogenesis Dysfunction in Alzheimer’s Disease

**DOI:** 10.3390/ijms221910744

**Published:** 2021-10-04

**Authors:** Bolanle Fatimat Olabiyi, Catherine Fleitas, Bahira Zammou, Isidro Ferrer, Claire Rampon, Joaquim Egea, Carme Espinet

**Affiliations:** 1 Molecular Developmental Neurobiology Group, IRBLleida-UDL Rovira Roure 82, 25198 Lleida, Spain; olabiyibola@gmail.com (B.F.O.); catherine.fleitas@gmail.com (C.F.); zammou.bahira@gmail.com (B.Z.); joaquim.egea@udl.cat (J.E.); 2 Institute of Molecular Psychiatry, Medical Faculty, University of Bonn, 53127 Bonn, Germany; 3 Departament de Patologia i Terapèutica Experimental, Universitat de Barcelona, 08007 Barcelona, Spain; 8082ifa@gmail.com; 4 Centro de Investigación Biomédica en Red de Enfermedades Neurodegenerativas (CIBERNED), Hospitalet de Llobregat, 08900 Barcelona, Spain; 5 Centre de Recherches sur la Cognition Animale (CRCA), Centre de Biologie Intégrative (CBI), Université de Toulouse, CNRS, UPS, 31062 Toulouse, France; Claire.Rampon@univ-tlse3.fr

**Keywords:** Alzheimer’s disease, adult neurogenesis, pro-NGF, p75, dentate gyrus, memory impairment

## Abstract

In recent decades, neurogenesis in the adult brain has been well demonstrated in a number of animal species, including humans. Interestingly, work with rodents has shown that adult neurogenesis in the dentate gyrus (DG) of the hippocampus is vital for some cognitive aspects, as increasing neurogenesis improves memory, while its disruption triggers the opposite effect. Adult neurogenesis declines with age and has been suggested to play a role in impaired progressive learning and memory loss seen in Alzheimer’s disease (AD). Therefore, therapeutic strategies designed to boost adult hippocampal neurogenesis may be beneficial for the treatment of AD. The precursor forms of neurotrophins, such as pro-NGF, display remarkable increase during AD in the hippocampus and entorhinal cortex. In contrast to mature NGF, pro-NGF exerts adverse functions in survival, proliferation, and differentiation. Hence, we hypothesized that pro-NGF and its p75 neurotrophin receptor (p75NTR) contribute to disrupting adult hippocampal neurogenesis during AD. To test this hypothesis, in this study, we took advantage of the availability of mouse models of AD (APP/PS1), which display memory impairment, and AD human samples to address the role of pro-NGF/p75NTR signaling in different aspects of adult neurogenesis. First, we observed that DG doublecortin (DCX) + progenitors express p75NTR both, in healthy humans and control animals, although the percentage of DCX+ cells are significantly reduced in AD. Interestingly, the expression of p75NTR in these progenitors is significantly decreased in AD conditions compared to controls. In order to assess the contribution of the pro-NGF/p75NTR pathway to the memory deficits of APP/PS1 mice, we injected pro-NGF neutralizing antibodies (anti-proNGF) into the DG of control and APP/PS1 mice and animals are subjected to a Morris water maze test. Intriguingly, we observed that anti-pro-NGF significantly restored memory performance of APP/PS1 animals and significantly increase the percentage of DCX+ progenitors in the DG region of these animals. In summary, our results suggest that pro-NGF is involved in disrupting spatial memory in AD, at least in part by blocking adult neurogenesis. Moreover, we propose that adult neurogenesis alteration should be taken into consideration for better understanding of AD pathology. Additionally, we provide a new molecular entry point (pro-NGF/p75NTR signaling) as a promising therapeutic target in AD.

## 1. Background

The neurodegenerative process involved in Alzheimer’s Disease (AD) pathogenesis often begins with memory loss and results in defects in multiple cortical activities later on. It is no longer new that adult neurogenesis occurs in the human brain and contributes to the cognitive functioning of the hippocampus [1,2]. Empirical evidence has shown that adult neurogenesis declines with age and that alteration of the neurogenic process contributes to the cognitive deterioration and learning difficulties and memory loss in AD [3,4,5,6].

In the mouse, adult-generated neurons are formed in two main brain regions: the subgranular zone (SGZ) located in the dentate gyrus of the hippocampus, and the subventricular zone (SVZ) of the lateral ventricles, which contains lateral, medial, and dorsal microdomains [7]. In the hippocampus, new functional neural cells arise from the adult neural stem cells (NSCs) via the amplification of intermediate progenitors and neuroblasts, followed by the integration of these new neurons into existing neural circuits [8].

Hippocampal neurogenesis has received considerable attention over recent decades because of its role in learning and memory [9,10] and because during AD it has been shown that hippocampal adult neurogenesis is greatly impaired and significantly contributes to learning and memory deficits associated with this pathology [2,3,4,11].

During adult neurogenesis, newly born cells express distinct neurogenic markers characteristic of the different stages of the process. The Primary Neural Progenitor cells (type 1 cells) are located in the subgranular cell layer (SGL) of the DG, where they slowly divide and express stem cell markers such as nestin and Sox2. They then give rise to intermediate Neuronal Progenitor Cells (type 2 cells), which are more proliferative and have only short processes. Type 2 cells are categorized into type 2a and 2b cells; type 2a cells express stem cell markers and type 2b cells express immature neuronal markers such as doublecortin (DCX), and they in turn give rise to more mature neurons, expressing first calretinin+ and then calbindin protein, reflecting their complete differentiation into granular neurons. During the second week after cell division, new-born neurons begin to extend their axonal processes to the CA3 region of the hippocampus and their dendritic processes towards the molecular layer [8]. At this stage, electrophysiological recordings show that they receive direct, slow GABAergic synaptic input, and start to show spines on dendrites. Between 4 and 6 weeks after birth, these neurons become fully mature and integrated into the circuit [12,13,14]. The decline of adult hippocampal neurogenesis observed with aging and to a greater extent in age-related pathological conditions such as AD suggests that neurogenesis plays a crucial role in proper brain functioning, including learning and memory (2). Thus, practical approaches to boost adult neurogenesis during neurodegeneration may be a promising strategy to combat the progressive loss of neurons and related cognitive dysfunction associated with AD. For this, it is essential to know the exact molecular mechanisms involved in the loss of adult neurogenesis during AD.

Neurotrophins are a broad family of secreted growth factors that are important for the survival, growth, and maintenance of neurons, with a crucial role in synaptic plasticity, learning and memory [15,16]. Of particular interest are nerve growth factor (NGF) and brain derived neurotrophic factor (BDNF). Most neurotrophic factors are synthesized as precursor forms (pro-forms, such as pro-NGF or pro-BDNF) and undergo proteolytic cleavage to render the mature neurotrophic forms under physiological conditions, the mature form of neurotrophins is generally produced from the intracellular activities of proteases such as convertases to elicit their trophic functions (17). Numerous studies have shown that proneurotrophins may be secreted without being cleaved, and this has deleterious effects on neuronal function [5,6,17,18,19,20]. The pro and mature forms of these neurotrophins have different affinities and specificities for various receptors, such as the p75 neurotrophic receptor (p75NTR). p75NTR belongs to the TNF family (tumor necrosis factor) of death receptors but has no catalytic activity. It has multiple roles in cellular homeostasis, apoptosis, cell cycle arrest, survival and proliferation which depend on the ligand and co-receptor binding to p75NTR [21]. For instance, when bound to mature neurotrophins, p75NTR interacts with Trk receptors to promote cell survival [15,16]. On the other hand, when p75NTR binds by precursor forms of neurotrophins, such as proNGF and proBDNF, it interacts with sortilin to form p75NTR-sortilin receptor complex, which trigger several death signaling pathways. These include NRAGE and NRIF and JNK kinases [21,22,23] or the processing of p75NTR intracellular domain (ICD) by two secretases, α and γ, releasing the 20 kDa ICD fragment (p75ICD) which interacts with TRAF6 and NRIF and translocate to the nucleus to facilitate the expression of proapoptotic genes [18,24,25,26,27]. 

Interestingly, several studies have implicated the precursor forms of the neurotrophins in many aspects of the pathogenesis of AD, especially in cell death [17,18,19,24]. For instance, in a previous study, we described how intra cerebro ventricular (ICV) injection of proNGF elicited spatial memory deficits in control mice, similar to those observed in AD mouse models such as the APP/PS1 mice [18,19]. This effect is probably exacerbated by the observation that under pathological conditions such as in AD, there is an increase of oxidative stress that may affect proneurotrophins (by undergoing advanced end glycation products (AGE)-derived modifications) that prevent proteolytic cleavage and maturation thus impairing cell survival and facilitating cell death [18,19,24]. However, it remains unclear whether the pro-neurotrophin/p75NTR signaling is involved in other processes beyond cell death, related to the AD pathology. In the present study, we investigated the contribution of the pro-NGF/p75NTR signaling pathway in adult hippocampal neurogenesis in AD patients and in APP/PS1 transgenic mouse model for AD and its impact in the cognitive dysfunctions of the disease. In AD models, the percentage of DCX+ progenitor cells in the DG is reduced. We found that p75NTR is expressed in these DCX+ cells in both controls and AD. However, the levels of p75NTR in DCX+ progenitor cells are significantly decreased, suggesting a role of p75NTR signaling in adult neurogenesis in AD. Interestingly, injection of neutralizing antibodies against proNGF restored the memory loss of APP/PS1 AD mouse model and increase the percentage of DCX+ progenitor cells in the DG of these animals.

## 2. Methods

### 2.1. Human Brain Samples

Brain samples were obtained from the Institute of Neuropathology, Bellvitge University Hospital. Brain tissues were obtained from the Institute of Neuropathology HUB-ICO-IDIBELL Biobank and the Hospital Clinic-IDIBAPS Biobank following the guidelines of Spanish legislation on this matter (Real Decreto de Biobancos 1716/2011) and approval of the local ethics committees (Table 1 and Table 2). For autopsy, one hemisphere was rapidly cut in 1 cm thick coronal sections, and selected areas of the encephalon were dissected, frozen on dry ice, and stored at −80 °C in labelled plastic bags until use. The other hemisphere was fixed by immersion in 4% buffered formalin for three weeks for morphologic examination. The neuropathological study was carried out on twenty-five regions of the cerebral cortex, diencephalon, thalamus, brainstem, and cerebellum. De-waxed paraffin sections were stained with haematoxylin and eosin and Klüver-Barrera, then processed for immunohistochemistry of microglia-specific markers, glial fibrillary acidic protein, β-amyloid, phosphorylated tau, α-synuclein, TDP-43, ubiquitin, and p62. Neuropathological diagnosis of AD was carried out following the Braak and Braak stages [28] adapted to paraffin sections [29]. Cases with concomitant pathologies, including Lewy body diseases, tauopathies (particularly argyrophilic grain disease), vascular diseases, TDP-43pathies, and metabolic syndrome were excluded. Control and disease cases were processed in parallel. The anterior hippocampus area was used for further immunohistochemical studies.

### 2.2. Animal Model

Considering that amyloid precursor protein (APP) and Presenilin 1 (PS1) are mutated in 78% and 18% of the familial cases of Alzheimer, respectively, the double transgenic APP/PS1 mouse model has been one of the most commonly used models in the study of Alzheimer’s disease [30,31,32].

APPswe/PS1dE9 with a C57BL/6 background (double transgenic mice expressing a chimeric mouse/human amyloid precursor protein and a mutant human PS1 with deletion in exon 9) were purchased from the Jackson Laboratory and kept in a specific pathogen-free environment under standard animal housing conditions in a 12 h dark-light cycle with free access to food and water in the animal house facility of the Universitat de Lleida. Heterozygous males were bred with wild-type C57BL/6 females. Animal procedures were conducted according to ethical guidelines (European Communities Council Directive 86/609/EEC) and approved by the local ethics committee of the Universitat de Lleida. For experiments, tail biopsies were taken from P0 offspring for genotyping by PCR according to the PCR conditions suggested by the Jackson Laboratory. Mice not expressing the transgene were used as controls. All the experiments are performed using 50% males and 50% females on average.

### 2.3. Behavioural Test: Morris Water Maze

The experimental animals were subjected to Morris water maze (MWM) test to assess their cognition/memory associated with hippocampal function [33,34]. All of the behavioral procedures were conducted at the same time of day, in an isolated room every day for 4 days. The tank was divided into four virtual quadrants containing one visible and different extra-maze cue each. The cues (black 4 × 4 cm geometric forms) were located at 20 cm from the water in the white circular extra-maze wall. A circular scape platform (diameter 9 cm) was located in the center of one quadrant (target quadrant) and hidden under the opaque water surface (23.5 °C–0.5 °C), in a water tank of 150 cm diameter and 30 cm high. The platform remained at a fixed location during training. The three other quadrants (opposite, adjacent 1 and 2) contained the starting points which were used in a pseudo-randomized order that varied across blocks of training trials. The mouse had to find the invisible platform using the extra-maze cues. Four trials per day with start positions close to the four geometrical signs were carried out, and latency in reaching the platform was recorded. Cut-off time to find the platform was 90 s, and mice failing to find the platform were placed on it and left there for 15 s. Each trial for a single animal was 30 min apart from the previous [18].

### 2.4. Stereotaxic Injection

Eight to nine-month old female and male APPsw695/PS1dE9 mice and control animals were placed under isoflurane anesthesia for a couple of minutes, after which they were injected intraperitoneally with the analgesia Rompun (2%) and ketamine (10%) in 0.9% NaCl. Then, mice were fixed securely unto the stereotaxic apparatus, and the skull was carefully exposed. The stereotaxic apparatus was then adjusted to the accurate position after which a metal bar driller was slowly used to make a hole in the skull. A volume of 5 µL per DG of the anti-proNGF antibody [17] or BSA control solution was gradually injected bilaterally into the dentate gyrus according to the stereotaxic coordinates (Bregma: anteroposterior: 2 mm, lateral: +/− 1.6 mm, dorsolateral: 2.5 mm from the skull), at a speed of 1 µL/min. After the successful injection, the needle was held in place for another minute before it was retracted slowly at the same rate of 1 µL/min speed. After this, the incisions were stitched, and the mice were returned to their home cages for a recovery period of 10 days.

Another set of APP/PS1 transgenic mice were stereotaxically injected with enhanced green fluorescent protein (eGFP) expressing the Moloney leukaemia-derived retroviral vector pCMMP-IRES2eGFP-WPRE. This was carried out using the previously described protocol [3].

### 2.5. Immunohistochemistry

A week after the behavioral study, the mice were anesthetized with Rompun (2%, Bayer)/ (Ketamine 0.1 mg/g, Merial) and transcardially perfused with NaCl (9%) followed by 4% PFA in PBS. Brains were dissected and fixed overnight in 4% PFA in PBS, then were washed with PBS 3 times for 10 min and subsequently cryoprotected in 30% sucrose (Scharlau) in PBSO/N, embedded in cryoprotective tissue-freezing medium (General Data), and stored at −80 °C. A volume of 30 µM serial coronal sections (cryosections) were then made using a cryostat (Leica CM 3000, Wetzlar, Germany), and collected in Super frost PlusTM slides (ThermoFisher, Lenexa, KS, USA) and stored at −80 °C for further use.

The immunohistochemistry protocol followed in this study varied slightly according to the sets of samples analyzed: hippocampal slices obtained from blocking α-proNGF stereotaxically injected mice, GFP retroviral-injected mice, or human samples. Pictures at 40X were made from all the DG. Then we selected randomly the number of views enough to cover the 300 DAPI positive neurons. Positive cells are expressed as the average of the % positive on the number of DAPI cells in each field. Co-localization is expressed as % of p75 cells on the total of DCX positive cells in each field. The procedures are summarized as follows:

### 2.6. Immunostaining Protocol for Human Samples

Human samples were deparaffined by heating them to 62 °C for 30 min and then substituted in xylol 2 times at 10-min intervals. A series of ethanol washes were then done in the lamina flow hood at varying concentrations as follows: 2 times in ethanol 100%, 2 times in ethanol 95%, and 2 times in ethanol 60%; the interval between each of these washes was 5 min. The resulting samples were rinsed in distilled water for 10 min, followed by 3 10-min washes in PBS 1X and then 1 10-min wash in 50 mM NH4Cl+PBS1X. Lipofusion was done under black conditions to eliminate autofluorescence by blocking in black Sudan suspension for 25 min at room temperature before antigen retrieval (10-min wash in preheated Tris-HCl buffer 20 mM pH 9.5 at 97 °C for 20 min), after which it was cooled down. The next step was to wash it in 0.1% PBST 2 times for 15 min and block with 5% donkey serum for 1 h. Primary antibodies were then added at working concentration 1:100 and kept at 4 °C till the next day (rabbit DCX Santa Cruz, Santa Cruz, CA, USA; goat anti-p75ECD R and D, Minneapolis, MN, Canada). On the following day, having spent 28 h in the cold room, the samples were left at room temperature for 2 h before proceeding with the 3 washes in PBST 0.1% at 15-min intervals. The samples were then incubated with secondary antibodies (working dilution 1:10,000 plus DAPI in blocking solution) for 2 h. They were then washed 3 times in PBS Tween 0.1% and a last washing in 0.1% PBS before they were finally mounted with the coverslip using the mounting medium Fluoromount G (Southern Biotech 0100-01, Birmingham, AL, USA).

### 2.7. Immunostaining Procedure for Mouse Cryosections

The sections were consequently subjected to one-hour blocking and permeabilization process with PBST and 1% donkey serum (5%) (Jackson 017-000-121 IR), then incubated with primary antibodies (in blocking solution 1:100) (rabbit DCX Santa Cruz; goat anti-p75ECD R and D), and subsequently kept at 4 °C for 5 days. On the 5th day, the samples were removed from the cold room and kept at room temperature for 2 h. After that, they were washed three times with PBST 0.1% at 10-min intervals; and then incubated with secondary antibodies in blocking solution (Working dilution 1:10,000 plus DAPI for 2 h at room temperature) (donkey anti-goat Cy3, 1:10,000; donkey anti-rabbit Alexa488, 1:500; donkey anti-mouse Cy3, 1:10,000 (all from Jackson Immunoresearch, Philadelphia, PA, USA). Finally, the cells were washed three more times with PBST 0.1% for 15 min, after which they were mounted with coverslips using Fluoromount G (Southern Biotech 0100-01). Negative controls for secondary antibodies are done (Supplementary Figure S2).

### 2.8. Immunostaining Protocol for GFP Retroviral Injected Hippocampal Slices

The mouse hippocampal slices were subjected to immunohistochemistry protocol performed in batches in round-bottomed Eppendorf tubes. The slices were first placed at room temperature for 40 min, followed by 4 free-floating washes with PBST (0.25% Triton) of 5 min each. After that, they were washed 3 times in PBST (0.1%) at 10 min intervals and then in PBST0.1%+NH4Cl 50 mM for 10 min. Antigen retrieval was done using pre-heated citrate buffer and the antigens were then placed in 95 °C boiling water for 30 min. The slices were cooled for about 15 min and washed 2 times in PBST (0.1% Triton) to remove remains of the fixing solution. Then, they were incubated with the blocking solution following the protocol described in the section below but in batches in the Eppendorf tubes. The antibodies were used in blocking solution at 1:100 (goat anti-p75ECD R and D; rabbit anti-DCX Santa Cruz). After that, they were washed three times with PBST 0.1% at 10 min intervals, and then incubated with secondary antibodies in blocking solution (working dilution 1:10,000 plus DAPI for 2 h at room temperature). Finally, the slices were carefully transferred from Eppendorf tubes to super frost glass slides and mounted with coverslips using Fluoromount G (Southern Biotech 0100-01). Fluorescence images were acquired on a confocal microscopy setup (Olympus FV1000, 60X PlanApo using software Fluoview v.4.3) or on an inverted fluorescence microscope (OlimpusIX71, 20X LCPlanFl).

All microscopes were set to collect images below saturation and were kept constant for all images taken in one experiment.

### 2.9. Statistical Analysis

Statistical analysis of data was performed using SPSS statistical software (SPSS for Windows, v.16, SPSS, Inc., Chicago, IL, USA). Student’s *t*-test and ANOVA were used to compare quantitative with qualitative variables and χ2 test was used for categorical variables. We performed Bonferroni post-hoc t-tests to further investigate statistically significant ANOVA results. Values were expressed as mean ± SD, and statistical significance are indicated as follows; *p* ≤ 0.05 (*), *p* ≤ 0.01 (**) or *p* ≤ 0.001 (***).

## 3. Results

### 3.1. Human and Mouse Hippocampal NSCs Express p75NTR

The existence of adult neurogenesis in human DG samples and its decrease in AD have recently been evidenced [1]. The mechanisms regulating such a decrease in AD remains widely unknown. In previous studies, we and others reported a significant increase in pro-neurotrophins in the hippocampus of AD patients [5,6,17,18]. In contrast, the overall expression of p75NTR was high and not affected in the hileal region of the DG of AD patients, ([9] and data not shown). Similarly, the p75NTR co-receptor sortilin, involved in apoptotic signaling was ubiquitously expressed in human DG, and no changes in the expression pattern were detected in AD (Supplementary Figure S1).

**Figure 1:**
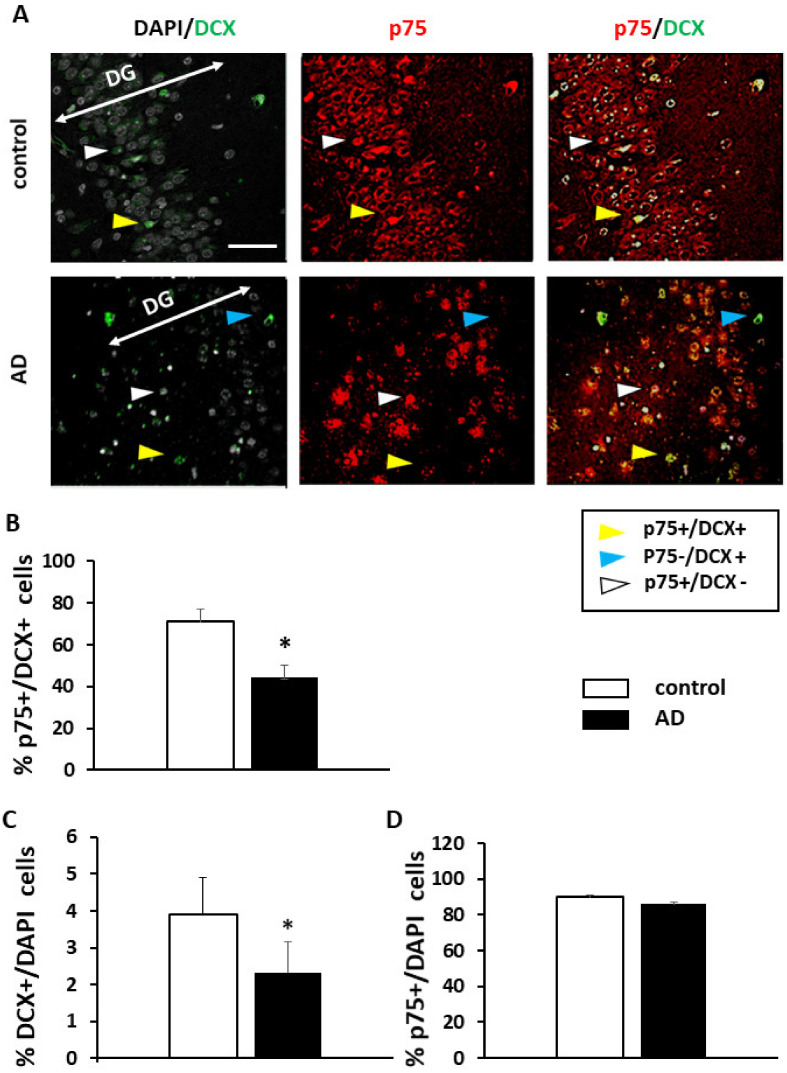
DCX and p75NTR expression in the human DG. (**A**) Representative confocal images of the neurogenic positive cells for DCX (green), p75NTR (red), and DAPI (grey) in DG of human samples of AD and control patients. Control shows greater colocalization of DCX and 75 than AD samples. Example of colocalization of DCX and p75 compared to AD samples (yellow arrow), DCX+ and p75- cells (blue arrow) and DCX-and p75+ cells (white arrow). Scale bar = 50 μM. Bars show the quantification of p75 and DCX colocalization, expressed as the mean of % of p75NTR+ on the DCX + cells (**B**) % DCX+/DAPI cells (**C**) and % p75+/DAPI cells (**D**). Enough pictures at 40X were randomly selected to cover 300 DAPI positive neurons (**C**). AD shows a decreased colocalization of DCX and p75 with respect to controls. (*n* = 3 control, *n* = 4 AD). *p* ≤ 0.05 (*).

**Figure 2:**
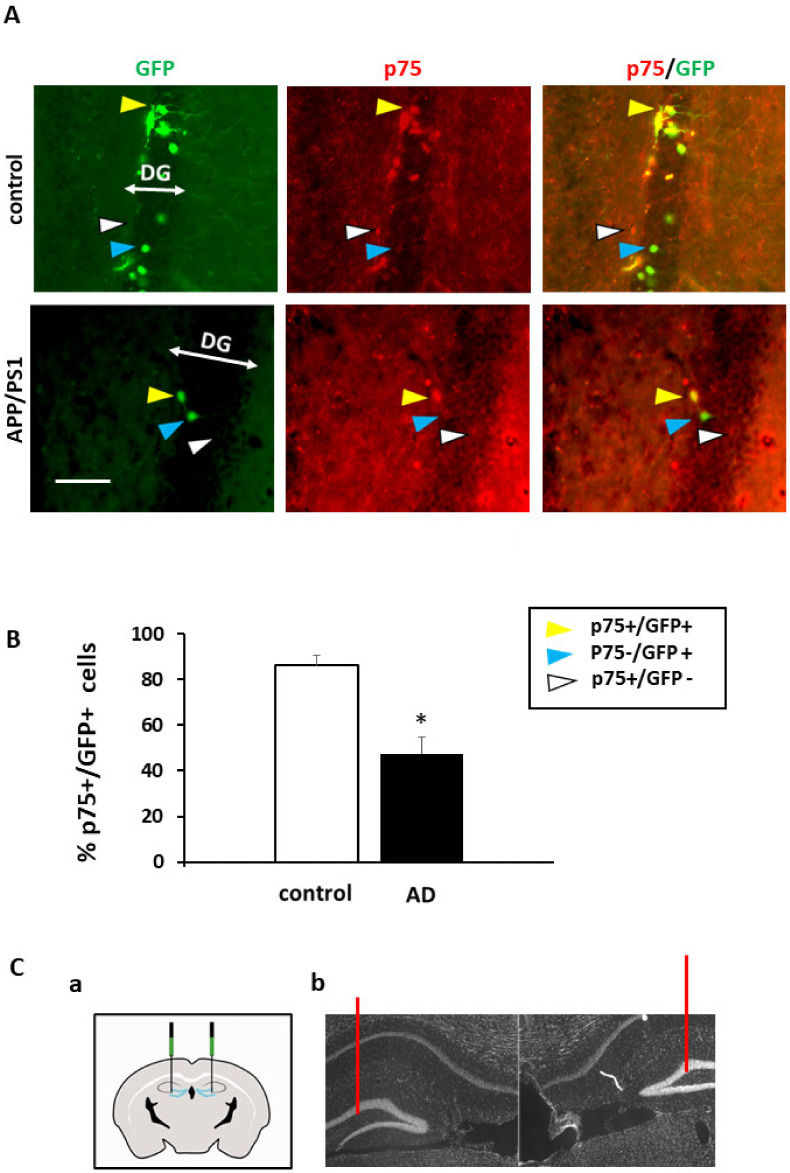
p75NTR expression in the DG of GFP retroviral injected mice. (**A**) Confocal images of the neurogenic cells transduced by GFP at 21 dpi, GFP+ cells (green) expressing p75NTR (red), p75NTR and GFP colocalization (merge). Example of colocalization of DCX and p75 compared to AD samples (yellow arrow), DCX+ and p75- cells (blue arrow) and DCX-and p75+ cells (white arrow). Scale bar = 10 μm; (**B**) Bars show decreased co-localization of GFP and p75 in APP/PS1 model as compared to control. Bars are expressed as the mean of % of p75NTR+ on the DCX+ cells (p75/DCX) in the DG of APP/PS1 mice and controls. Positive cells are considered those with staining in the cell body. Enough pictures at 40× were randomly selected to cover 300 DAPI positive neurons (*n* = 5 mice per group); (**C**) Histological map of DG injection (**a**), DAPI staining of hippocampal region, showing injection sites in the DG (bars in red) (**b**). *p* ≤ 0.05 (*), *p* ≤ 0.01.

In the present work we studied more in detail the expression of p75NTR in DG progenitor cells in order to determine whether the apoptotic effect of pro-neurotrophins, could account for the decrease of NSCs in human DG of AD subjects. First, we quantified the number of DCX+ progenitor cells and, as expected, there was a significant reduction compared to controls in AD patients (C 4.2 +/− 0.6; AD 2.2 +/− 0.7; T test *p* = 0.03, F 0.9 (Figure 1B). The number of p75NTR positive cells in DG, did not show significant differences between control and AD samples (T test *p* = 0.2, F 0.3) (Figure 1D). Next, we quantified the co-expression of p75NTR in DCX+ cells and observed that p75NTR and DCX are co-expressed in the control DG human samples to a substantial extent (71 ± 5.78%) (Figure 1A,B). In contrast, in AD samples, p75NTR and DCX co-expression was significantly reduced (44.25 ± 6.1%; T test *p* = 0.01, F 1.03) (Figure 1A,B). These changes of p75NTR expression in AD progenitors were suggestive of a role of p75NTR as signaling apoptotic death in newly generated neurons in the AD context. Next, we used the AD mouse model APP/PS1 and retroviral GFP delivery to specifically label NSCs in both, control and APP/PS1 mice. After 21 dpi (days post-injection), new neurons in the DG are intermediately differentiated and still expressing DCX [14]. At this time-point, immunohistochemistry against GFP and p75NTR was carried out to determine the extent of p75NTR+/GFP+ co-expression. In this model, the proportion of GFP+ neurons in the DG is significantly reduced (T test *p* = 0.01, F 10). We observed that most of the GFP+ cells expressed p75NTR in the DGs of control mice (86 ± 4.5) (Figure 2A,B). However, and similar to human AD samples, this co-localization was strongly reduced in APP/PS1 mice (47.6 ± 7; T test *p* = 0.002, F 0.4) (Figure 2A,B). In our previous results we did not find significant differences in the overall expression of p75NTR in the DG of AD models [9]. The percentage of DCX+ cells in the DG is very low (less than 5% of granular cells in control mice), suggesting that differences of p75NTR expression in the progenitor cells get diluted when the entire tissue is analyzed. On the other hand, these results in human and mouse AD samples indicate a specific reduction of p75NTR expression in progenitor cells which is interesting as it suggests a role for p75NTR in neuroprogenitor cells in the AD context.

### 3.2. Injection of proNGF Blocking Antibody Enhances Memory Function in Transgenic APP/PS1 Mice

**Figure 3:**
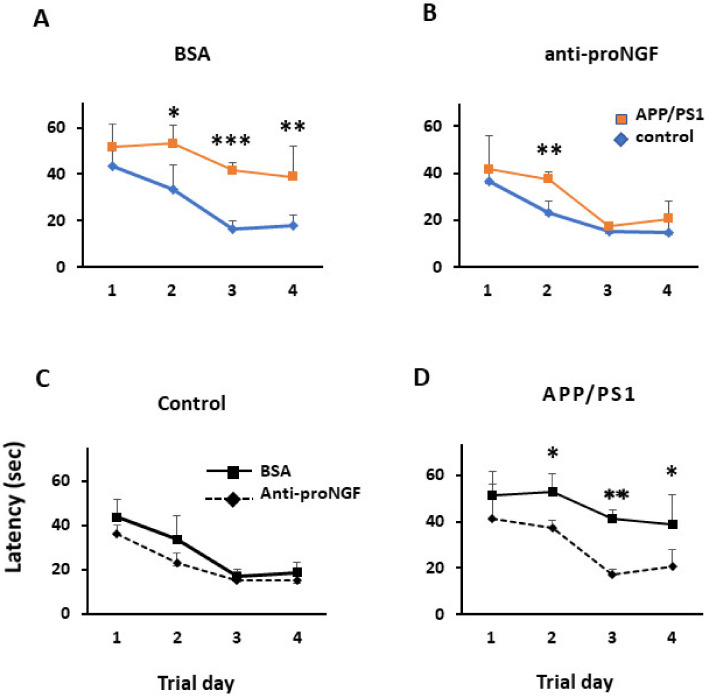
The injection of anti-proNGF antibody recovers the memory impairment in APP/PS1 mice (**A**) Morris water maze test in animals injected with BSA. Results show a higher latency in finding the hidden platform (latency (sec)) in the APP/PS1 transgenic mice (red line) compared to the control animals (blue line); (**B**) Morris water maze test in animals injected with anti-proNGF antibody. Results show a similar latency (sec) in the APP/PS1 transgenic mice (red line) compared to control animals (blue line); (**C**) Morris water maze test in control animals injected with BSA (solid line) or with anti-proNGF antibody (dashed line). Results show a similar latency in control animals injected either with BSA or with anti-proNGF antibody; (**D**) Morris water maze test in APP/PS1 animals injected with BSA (solid line) or anti-proNGF antibody (dashed line). Results show a significantly higher latency (sec) in the APP/PS1 transgenic mice when injected with the antibody (dashed line) compared to the injected with BSA. (*n* = 5 mice per group). *p* ≤ 0.05 (*), *p* ≤ 0.01 (**) or *p* ≤ 0.001 (***).

Behavioral and memory alterations are well described in APP/PS1 mice as disease progresses [30]. We have previously demonstrated that some of these cognitive defects (including spatial memory) and morphological changes (increase of apoptosis), could be recapitulated in control mice by a single ICV injection of proNGF, either purified from human AD-affected hippocampus or recombinant protein modified in vitro by AGE [18]. These observations, together with changes in p75NTR expression in DG progenitor cells in AD condition, let us to hypothesize that proNGF might have an important role during the development of the disease. To test this possibility, we wanted to examine whether ablation of proNGF in the DG of APP/PS1 mice could abolish their spatial memory deficits. For this, we run a Morris water maze (MWM) test which will allow to determine the effect of blocking proNGF activity on adult hippocampal neurogenesis and memory. Thus, we injected these mice and a control group with either anti-proNGF neutralizing antibodies (5 µg/DG) or BSA control solution (5 µg/DG) bilaterally in the DG, after which the mice were allowed to fully recover from the injection. The amount of anti-proNGF antibody that we injected in the present study should be enough to block the increase in proNGF found in the hippocampus of APP/PS1 animals. Then we subjected the animals to a MWM test and calculated the time in finding the hidden platform (latency). As expected, APP/PS1 group of mice injected with BSA solution, clearly display a memory delay similar over the days of training (difference between day 1 and 4 is not significant, ANOVA *p* = 0.15) compared to the control group which rapidly decrease the latency over the days of training indicating learning to find the platform (*p* = 0.0003) (Figure 3A). Noticeably, injection of anti-proNGF antibody in APP/PS1 mice strongly reduced time latency and the transgenic mice performed almost as good as the control group over the time of training (Figure 3B) (difference between day 1 and 4, ANOVA *p* = 0.0001). For the control group, no significant differences were observed between injection with anti-proNGF or BSA (Figure 3C). The ANOVA analysis gave the following parameters between variables: interaction F (1, 16) = 19.36, *p* = 0.0004; Time Factor F (1, 16) = 92.08, *p* < 0.0001 and treatment factor (BSA, proNGF) F (1, 16) = 71.77, *p* < 0.0001.

Taken together, our results suggest that memory loss of APP/PS1 mice is at least partially mediated by pro-NGF as injection of anti-pro-NGF significantly improved the memory tasks of these animals.

### 3.3. Injection of proNGF Blocking Antibody Enhances Neurogenesis in Transgenic APP/PS1 Mice

**Figure 4:**
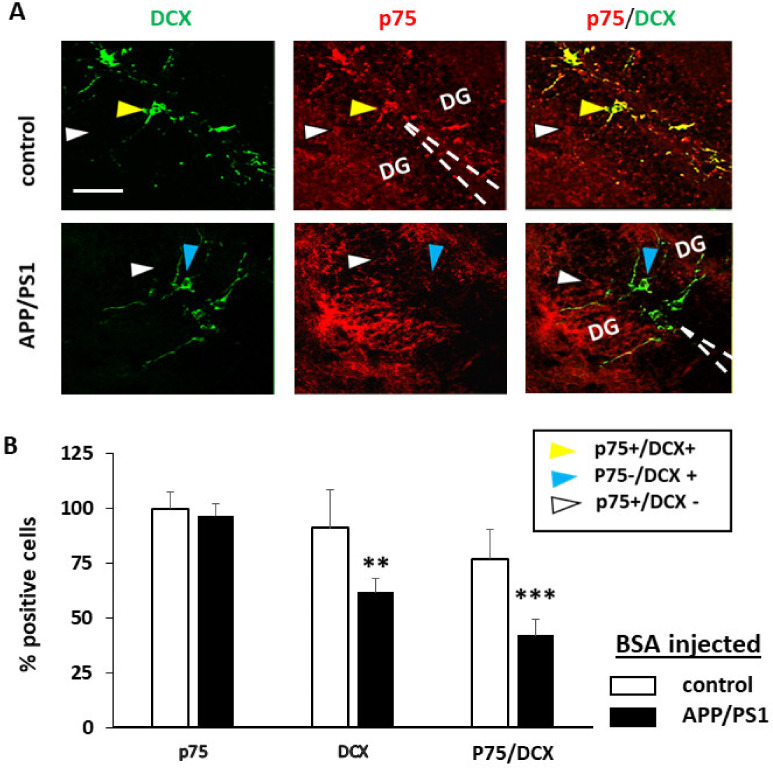
Neurogenesis in the DG of control and APP/PS1 mice in animals injected with control BSA. (**A**) Confocal images representing immunofluorescence pictures showing colocalization of p75 and DCX in the control and AD groups. p75 (red), DCX (green). Example of colocalization of DCX and p75 compared to AD samples (yellow arrow), DCX+ and p75- cells (blue arrow) and DCX-and p75+ cells (white arrow). Control showing greater colocalization of DCX and p75 than the APP/PS1 transgenic mice. Scale bar = 10 μm; (**B**) Bars represent the mean of % positive cells ± SD respect to total cells (p75NTR and DCX) or % of p75NTR+ on the DCX positive cells (p75/DCX) in the DG of APP/PS1 mice and controls. Positive cells are considered those with staining in the cell body. Enough pictures at 40× were randomly selected to cover 300 DAPI positive neurons. Values are expressed as the percentage of the highest mean (*n* = 5 mice per group). *p* ≤ 0.01 (**) or *p* ≤ 0.001 (***).

**Figure 5:**
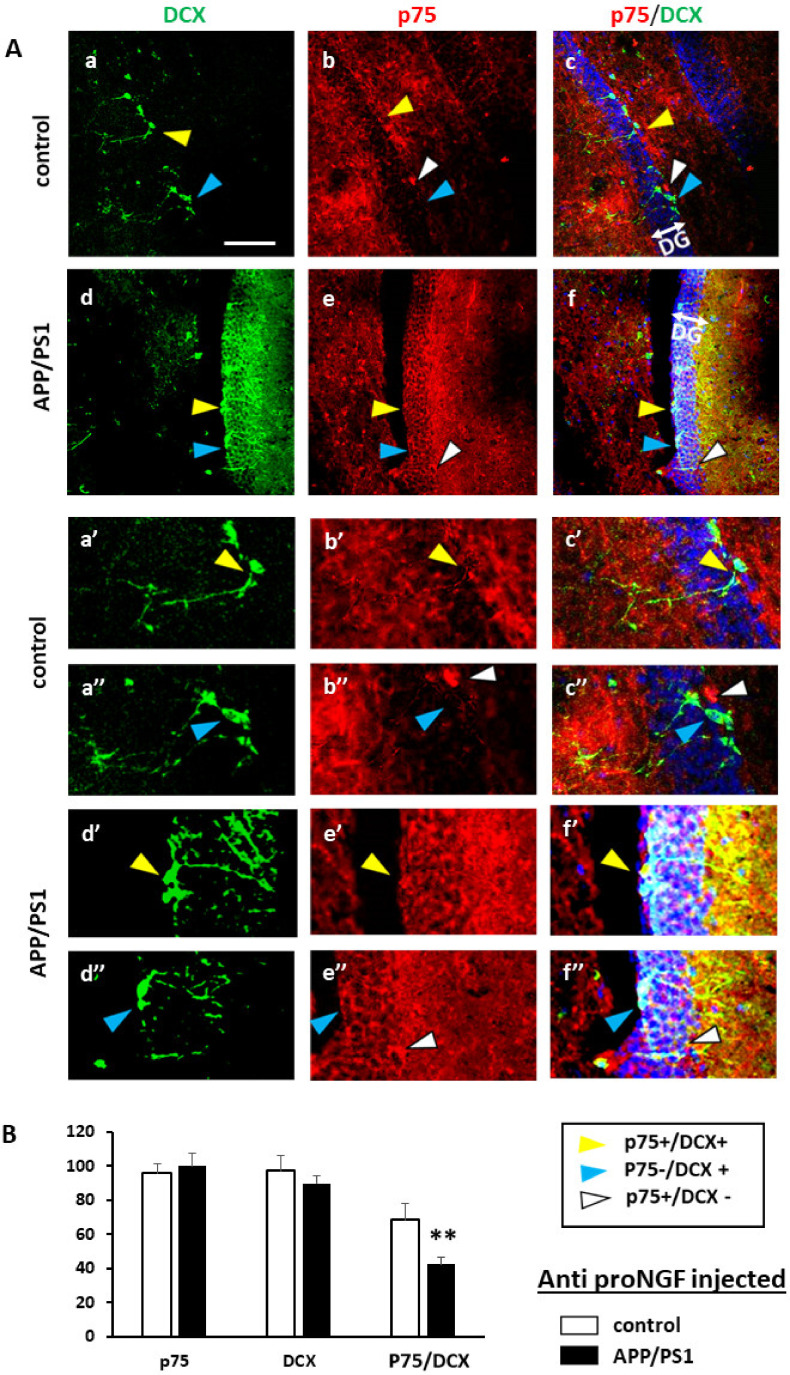
Neurogenesis is increased in the DG of APP/PS1m mice injected with anti-proNGF antibody. (**A**) Confocal images representing immunoflurorescence pictures showing colocalization of p75 and DCX in the control (**a**–**c**) and AD (**d**–**f**) groups. DAPI (blue), p75 (red), DCX (green). Controls showing greater colocalization of DCX and p75 than APP/PS1 transgenic mice. Example of colocalization of DCX and p75 compared to AD samples (yellow arrow), DCX+ and p75- cells (blue arrow) and DCX-and p75+ cells (white arrow). Scale bar = 15 μm. Pictures with quotation marks correspond to amplifications of the corresponding letters; (**B**) Bars represent the mean of % positive cells ± SD respect to DAPI stained cells per field or % of p75NTR+ on the DCX positive cells (p75/DCX) in the DG of APP/PS1 mice and controls. Positive cells are considered those with staining in the cell body. Values are expressed as the percentage of the highest mean. Enough pictures at 40× were randomly selected to cover 300 DAPI positive neurons. (*n* = 5 mice per group). *p* ≤ 0.01 (**).

**Figure 6:**
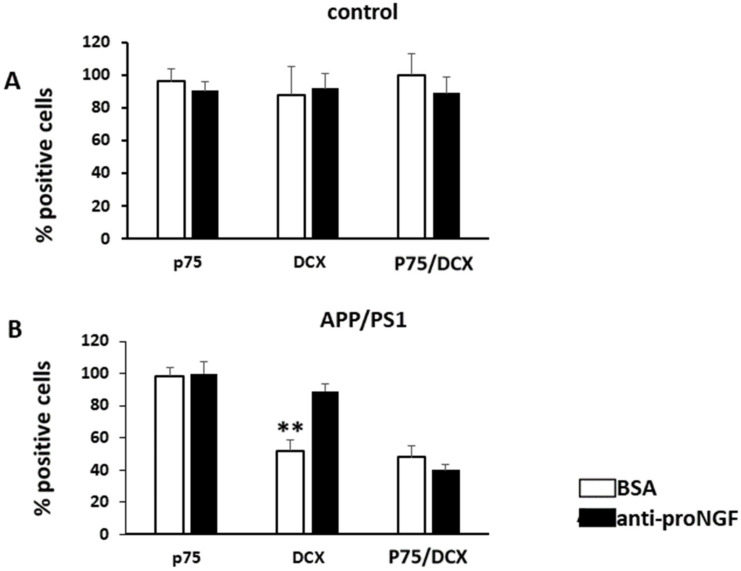
APP/PS1 animals show an increase in DCX positive cells after the injection of the anti-proNGF antibody. Comparison of BSA and anti-proNGF antibody injected into control mice (**A**) or into APP/PS1 mice (**B**). Quantification is refereed to Figure 5. Values are expressed as the percentage of the highest mean. (*n* = 5 mice per group). *p* ≤ 0.01 (**).

We next evaluated if anti-proNGF injection in APP/PS1 mice could have an effect on adult neurogenesis. For this, the animals used in the MWM test were sacrificed and the DG analyzed by immunofluorescence with specific antibodies against p75NTR and DCX. APP/PS1 mice showed lesser percentage of DCX+ cells (C 82% +/− 14, APP/PS1 61% +/− 6.2; T test *p* = 0.018, F 7.9) and, as we already observed in Figure 1, lower levels of p75NTR expression in DCX+ progenitor cells compared to controls, when injected with BSA (C 75% +/− 12, APP/PS1 37 +/− 7; *p* = 0.001, F 4.42) (Figure 4). As observed in Figure 1, expression of p75NTR in BSA injected DG, did not differ between control which is the reference number (100% +/− 7.2) and APP/PS1 (96% +/− 5.5) (T test *p* = 0.28, F 1.9). Interestingly, APP/PS1 mice injected with anti-proNGF, displayed similar percentage of DCX+ cells (90.3% +/− 4.5) as controls (98% +/− 8.2; *p* = 0.3, F 3.5) (Figure 5 and Figure 6B). No changes in percentage of positive cells for p75NTR (overall expression) (*p* = 0.19, F 0.39), for DCX (*p* = 0.42, F 0.8) or for p75NTR among the DCX+ progenitor cells (*p* = 0.25, F 0.48) were found after BSA or anti-proNGF injection in control mice (Figure 6A). However, in the AD mice model, the levels of p75NTR in DCX+ cells after the injection of the antibody (40% +/− 5.1) remained low and similar to BSA-injected animals (48 +/− 9.2; *p* = 0.19, F 0.35) (Figure 5A,B and Figure 6B).

These experiments demonstrate that inhibition of proNGF prevents the loss of DCX+ progenitor cells in APP/PS1 mice, thus improving neurogenesis in these animals. Moreover, our results suggest that decreasing proNGF in the DG of APP/PS1 animals allows the development of NSCs, mainly those expressing less p75NTR (Figure 5B). At this point, it is tempting to speculate that this improvement of adult neurogenesis is in part responsible for the gain of spatial memory observed in APP/PS1 mice treated with anti-proNGF (Figure 3; see discussion below).

## 4. Discussion

It has been noted over the years that high concentration of pro-neurotrophins significantly contribute to the pathogenesis of Alzheimer’s disease [5,6,17,18,19,35]. Among other factors, oxidative stress, inflammation, excitotoxicity, and altered adult hippocampal neurogenesis have been suggested to play a role in the progression of AD [1,5,24,36,37]. In this study, we investigated the impact of variations in proNGF signaling on adult hippocampal neurogenesis in a mouse model of AD as well as in human brain samples from AD patients.

It is now well known that neurogenesis occurs in the DG of adult humans and that the process persists but is affected in AD patients. During the multistep process of neurogenesis, DCX resides at the crossroads of new neuron formation. It has been demonstrated that the expression of this marker sends a neurogenic signal to immature neural progenitor cells and contribute to instruct them to fully differentiate into mature neurons. As such, DCX has been widely used for the study of neurogenesis [1,10]. The coexpression of DCX and p75NTR examined in this study is of great importance because the ambivalent role of p75NTR in cell survival/death signaling depending on the ligand and co-receptor bound to it.

As previously reported by our group [6,17,18,19], the ligands of the p75NTR/sortilin signaling pathway of apoptosis, proNGF and proBDNF, are in general, present in greater quantities in AD human brain and more significantly, in the DG and hilar region of the hippocampus. Therefore, the expression of p75NTR in NSCs of the DG suggest a role of the p75NTR signaling in adult neurogenesis in the context of AD. Indeed, our findings reveal that p75 co-expression with DCX is sharply decreased in the DG of AD patients compared to healthy controls. We hypothesized that the decrease in p75NTR/DCX colocalization in AD patients could be due to the death of the neurons initially expressing higher levels of p75NTR and therefore more sensitive to proneurotrophins. In other words, during AD progression, the marked decline of adult neurogenesis could be related to neuronal degeneration/death mediated by the proneurotrophin/p75NTR signaling pathway.

After several contradictory reports regarding whether or not adult neurogenesis occurs in humans, Moreno-Jiménez and colleagues nicely demonstrate that it does occur in human [1]. The present work supports their findings in providing further evidence of its occurrence in humans.

Consistent with the reduction in the number of neurons co-expressing p75NTR/DCX that we observe in AD patients, we report the same trend in the APP/PS1 mouse model of AD. The choice of our animal model was prompted by the fact that in familial AD, more than half of the cases have inherited mutations in the presenilin 1 secretase and amyloid precursor protein (APP) [38]. The deletion in exon 9 of PS1 coupled with defective APP has been identified as the significant genetic error associated with this form of AD.

Based on this, we studied the neural stem cells of the DG of these APP/PS1 mice. Our immunohistochemical work indicates that neural stem cells of APP/PS1mice show less co-expression of p75 and DCX, just as is the case in AD patient’s samples. The evidence of adult neurogenesis in mice is less controversial; numerous studies have already established its existence in mice [3,4,8]. Using a GFP-expressing retroviral vector, we screened for p75 expression in dividing neural stem cells of APP/PS1 mice. We found that less GFP-labelled cells expressed p75 in AD mice compared to their wild-type counterparts. Our inference from these three experiments boils down to neurogenesis as a significant process that is impaired in AD.

Here we provide evidence that neurogenesis plays a crucial role in maintaining the granular neurons population in the DG of healthy subjects. When this process is altered by neurodegeneration, it could lead to an acceleration of neuronal death via the action of the proneurotrophin/p75 pathway. Reduction of SOX2 and DCX positive cells in the DG from human affected by AD, was described in 2011 [39]. Preliminary results (Supplementary Figure S3) show a high degree of co-localization of SOX and p75. Although, SOX positive cells can derivate either to neurons or to glial cells. In this work we are mainly focused on the new neurons involved in memory which is reduced in AD and the markers of new neuronal precursors that we use are the retroviral injection of GFP using samples at 21 dpi and the GFP positive neurons.

Neurodegenerative diseases share common features including neuronal death and behavioral abnormalities. In Alzheimer’s disease, memory and cognitive deterioration pose a serious threat to the wellbeing of the aging population. We showed that AD mice exhibited lower cognitive function based on the results obtained from the Morris water maze test which have been used in previous studies [18] to monitor the cognitive behavior of animals injected with purified pro-NGF and also to assess their learning abilities [18,19,33,34,39]. Using a same mice model, other behavioral tests have been used [3,4] to confirm that the loss of cognitive function in the disease is linked to the reduction of the number and the maturation of NSCs in the DG. Moreover, several more detailed MWM tests would allow the calculation of learning index scores and other criterion [40] that could be correlated with immunofluorescence results. Behavioral studies in our model following more detailed methods will be the focus of future work.

The implications of the roles of proneurotrophins and the p75 signaling pathway in the pathogenesis of AD that emerged from previous findings in our lab and others finally prompted us to examine the possible effects of inhibiting proNGF activity on the cognitive function of AD mice. It has been recently reported that a single intra-hippocampal injection of anti proBDNF in mice, attenuates hippocampal-dependent Learning and memory dysfunction in a mice model of Sepsis-Associated Encephalopathy [41]. This is in line with what we had previously reported that some of cognitive defect’s characteristic of mice model of AD, could be recapitulated in control mice by a single ICV injection of proNGF [18]. In the present study we found that after injecting proNGF-blocking antibody into the DG of the APP/PS1 mice, their memory and proportion of NSCs were greatly enhanced. We suggest that proNGF is partly involved in causing the cognitive deficits in the APP/PS1 mice and in disruption of human adult neurogenesis. 

In conclusion, NSCs of the dentate gyrus of control human samples express DCX and this expression significantly decreases in AD patients. The NSCs express p75NTR which colocalizes with DCX to a greater extent in the control than in the AD brain. Neurogenesis is diminished in the NSCs of AD patients and APP/PS1 mice model. APP/PS1 mice experience behavioral abnormalities associated with cognitive deficits. Upon administration of proNGF-blocking antibodies, the number of NSCs and the cognitive function of the AD mice were both greatly enhanced. The proneurotrophin/p75 signaling pathway has a negative impact on cognitive function and adult hippocampal neurogenesis. Hence, our findings, further support the idea that adult neurogenesis could serve as an alternative therapeutic target in the pathogenesis of AD.

## Figures and Tables

**Table 1:** Summary of the patients from whom hippocampal samples were obtained and studied. C: control, AD: Alzheimer’s disease; A, B and C: Braak and Braak’s classification of AD stages depending on amyloid plaques; 0-V: Braak and Braak’s classification of AD stages depending on the distribution and number of neurofibrillary tangles.

N	Age	Gender	Diagnosis	Braak	Post-Mortem
				Stages	Delay/Hours
1	46	F	C	0	9
2	47	M	C	0	5
3	24	F	C	0	6
4	79	M	AD	V/B	5
5	82	F	AD	V/B	2
6	79	M	AD	V/C	7
7	85	F	AD	VI/C	12

**Table 2:** Demographic and clinical characteristics of the patients from whom CSF samples were collected.

	Case (*n* = 15)	C (*n* = 15)	*p*
Male	4 (26.7%)	5 (33.3%)	0.7
Years	73.5 ± 12.1	70.5 ± 7.1	0.6
Age schooling	11.3 ± 5.0	11.3 ± 2.2	0.2
MMSE	19.1 ± 6.0	28.1 ± 1.8	0
Family history			
Presenilin AD	2 (13.3%)	0 (0%)	0.14
EA > 65 years	3 (20.0%)	5 (33.3%)	0.4
Pathological history			
Hypertension	6 (40%)	8 (53%)	0.46
Diabetes	3 (20.0%)	2 (13.3%)	0.6
Hypercholesterolemia	2 (13.3%)	7 (46.7%)	0.04
Depression	2 (13.3%)	7 (46.7%)	0.04
CSF AD Biomarkers	pg/mL	pg/mL	
Amyloid β	385.6 ± 128.1	856.2 ± 204.3	0
Total Tau	614.8 ± 270.2	280.7 ± 103.9	0.01
PhosphoTau	84.5 ± 27.7	55.4 ± 17.9	0

## Data Availability

Data sharing is not applicable to this article as no datasets were generated or analyzed during the current study.

## Supplementary Materials

Supplementary materials can be found at https://www.mdpi.com/article/10.3390/ijms221910744/s1.

## Abbreviations

AD: Alzheimer’s disease; BSA: Bovine serum albumin; ICV: intra cerebro-ventricular; DCX: doublecortin; ND; neurodegeneration; NSCs: neural stem cells; P/S: penicillin/streptomycin; PBS: phosphate buffered saline; PBS-T: PBS with 0, 1% Triton X-100; PFA: paraformaldehyde; proNGF: precursor form of NGF; PS1: presenilin 1; p75NTR: p75 neurotrophin receptor; SD: standard deviation; TBS-T: 50 mM Tris, pH 8.0; 133 mM NaCl, 0.2 % Tween 20.
