# Supplementary material for: proNGF Involvement in the Adult Neurogenesis Dysfunction in Alzheimer’s Disease
Source: Int J Mol Sci. 2021 Oct 4;22(19):10744. doi: 10.3390/ijms221910744 (PMC8509282; doi:10.3390/ijms221910744)
Supplement: Supplementary file 1 [file ijms-22-10744-s001.zip › ijms-1375902-supplementary.pdf]

Suplementary data. Figure S1

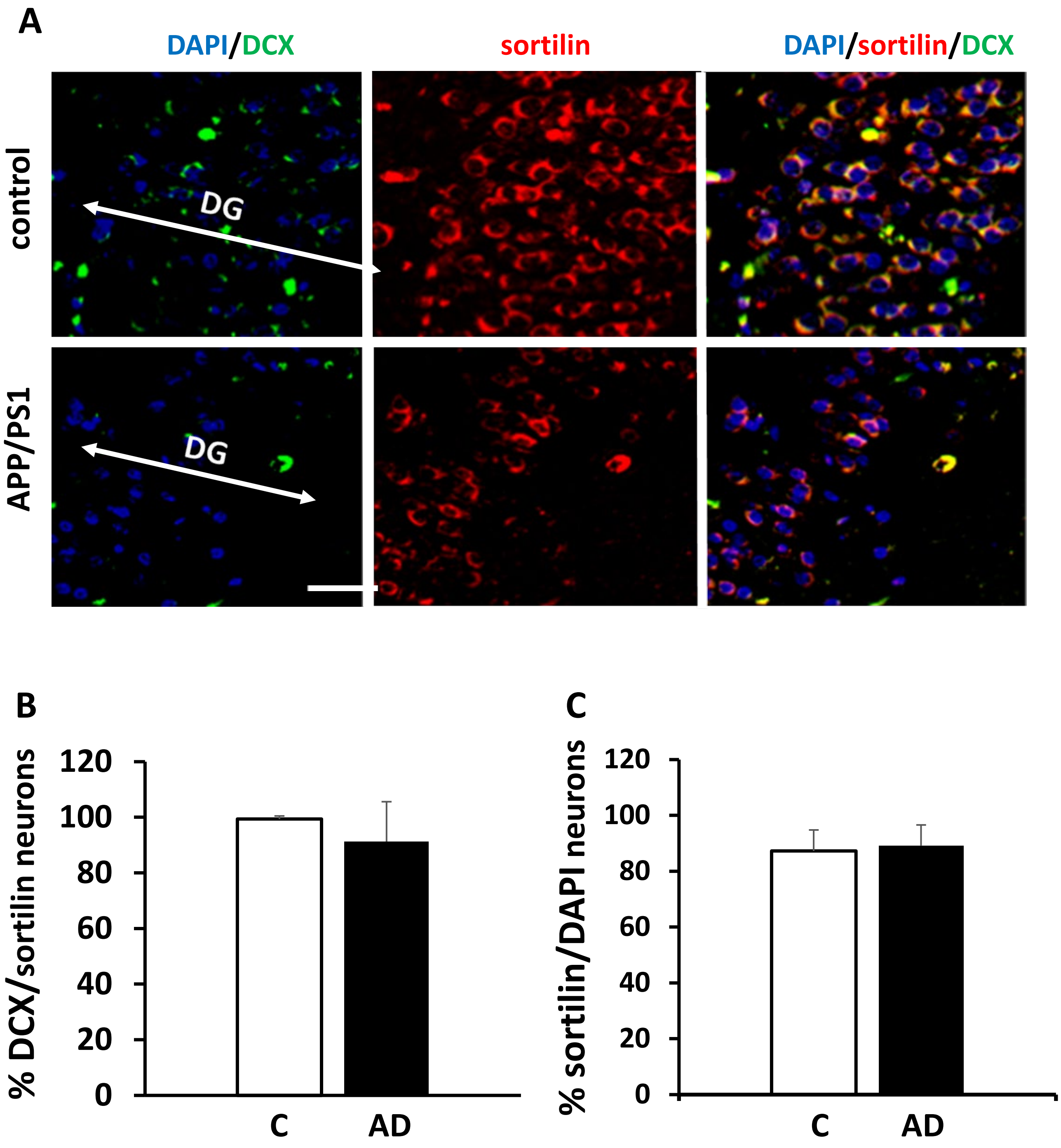

Figure S1. Sortilin expression in the DG of control and AD affected patients. (A) Confocal images of the DCX+ neurons (green) expressing sortilin (red), and DCX/sortilin colocalization. Scale bar = 50µm. Bars show no changes in co-localization of DCX and sortilin in APP/PS1 model as compared to control. Bars are expressed as the mean of % of p75NTR+ on the DCX positive cells (DCX/p75NTR) (B) and of sortilin/DAPI nuclei (C) in the DG of APP/PS1 mice and controls. Positive cells are considered those with staining in the cell body. 300 total cells (DAPI positives) from each sample are counted. Values are expressed as the percentage of the highest mean (n= 4 control, n= 5 AD).

Suplementary data. Figure S2

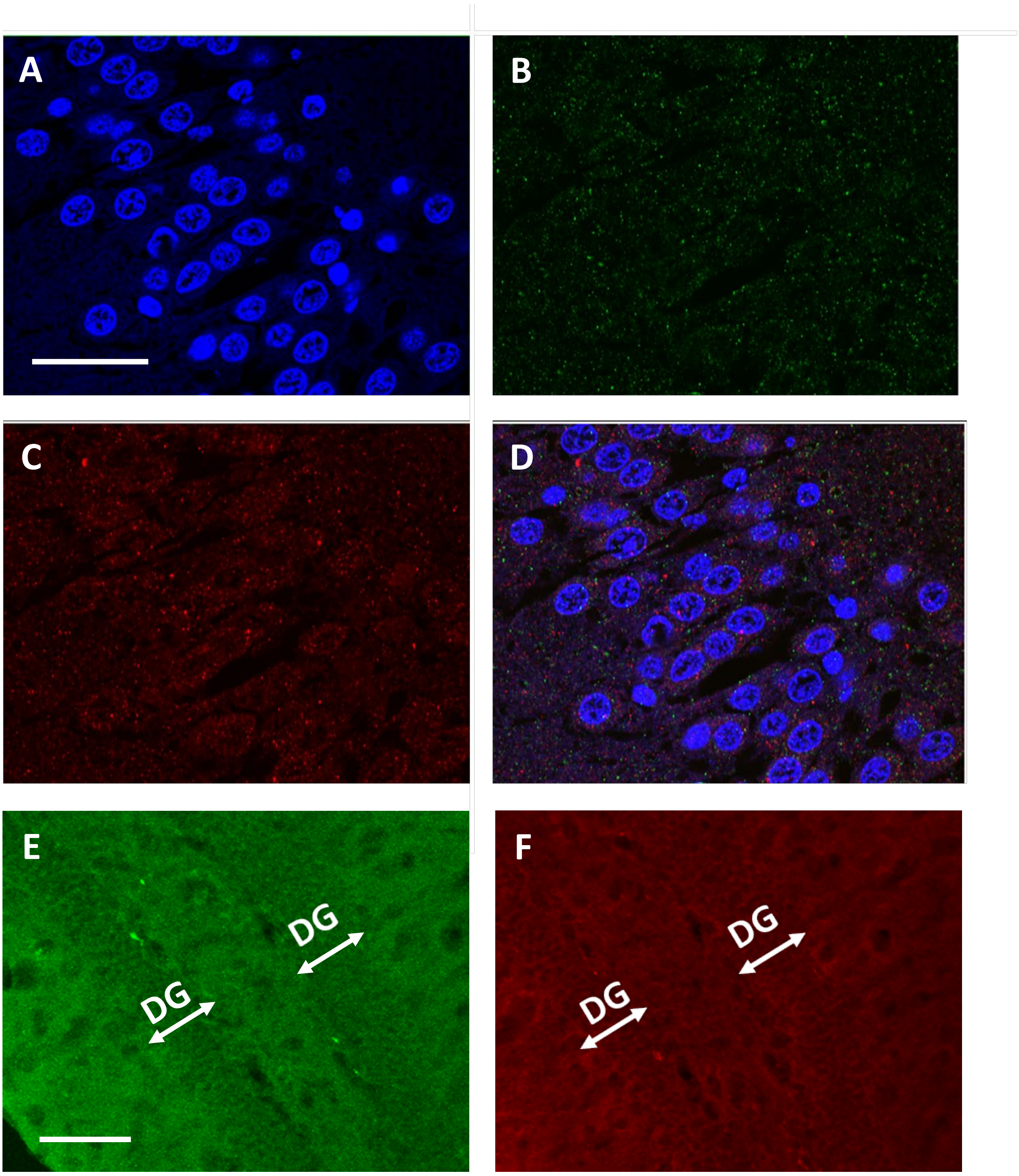

Figure S2. Negative controls of immunofluorescence. The figure shows the low level of autofluorescence or unspecific signal. Human DG from AD affected samples (A, B, C, D) incubated in the absence of primary antibodies. DAPI (A), Alexa488 (B), Cy3 (C), merge (D).APP/PS1 mice DG (E, F) incubated in the absence of primary antibodies. Alexa488 (E), Cy3 (F) following the procedure described in methods.

Suplementary data. Figure S3

A

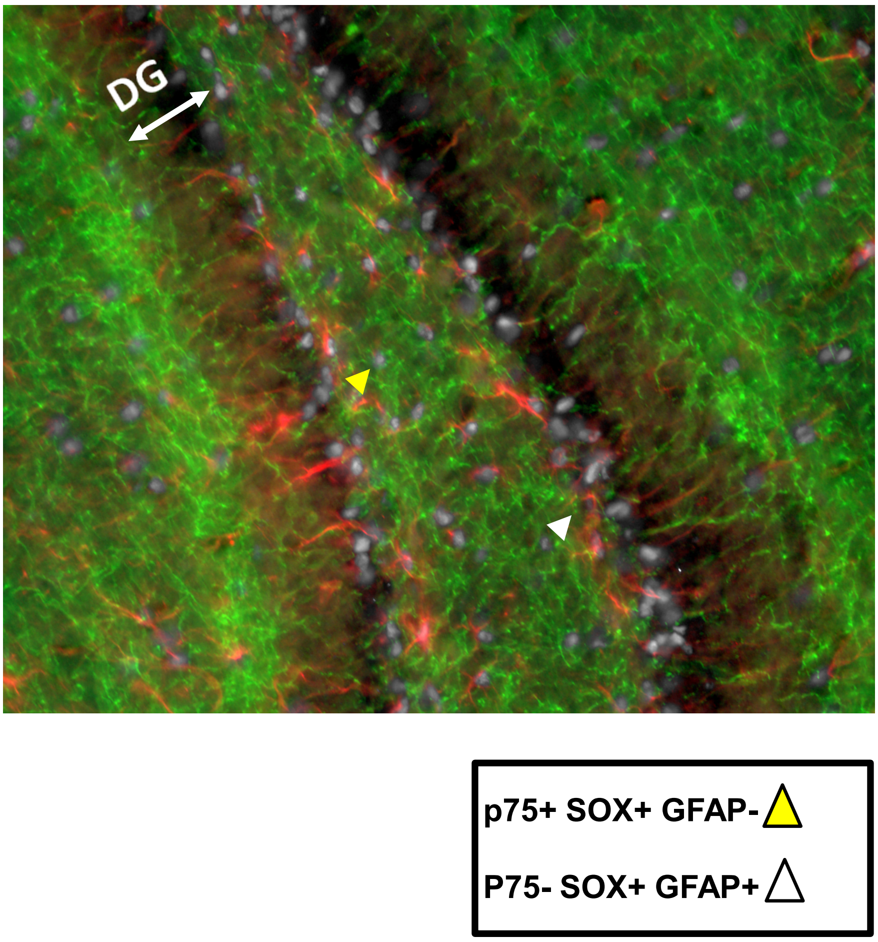

B

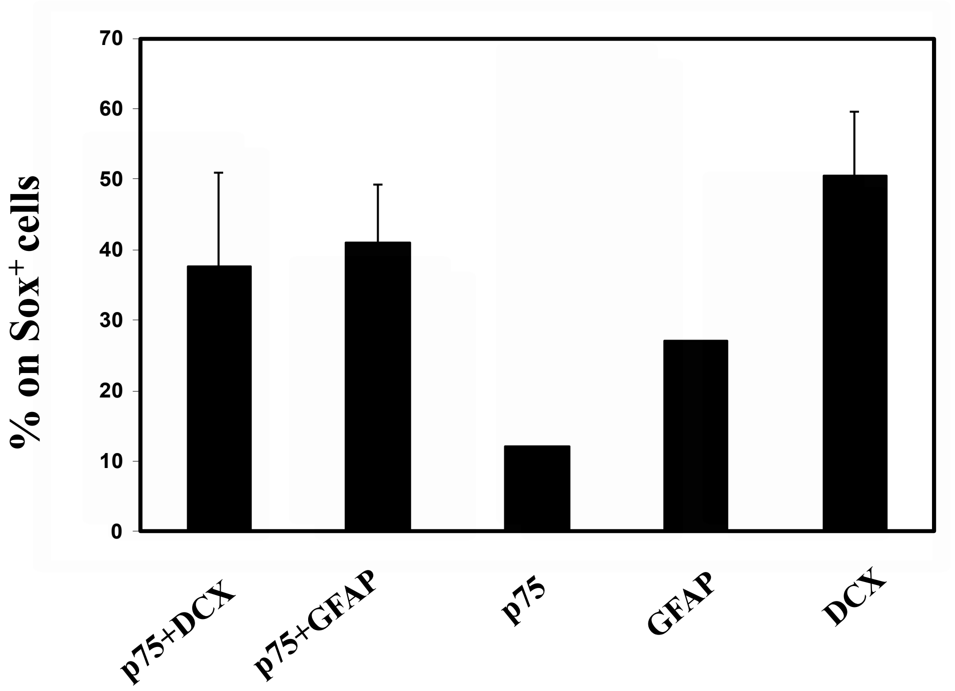

Figure S3. Colocalization of SOX2 and GFAP with p75.
(A) Representative immunofluorescence of adult male control mice with antip75 (extracellular domain) (green), SOX2 (white) and GFAP (red). Arrows indicate colocalization. (B) Quantification of the Positive neurons in the immunofluorescences. 300 total cells (DAPI positives) from each sample are counted.Triangles are representative positive cells as indicated in the legend. (n=2)
